# Disease Recurrence—The Sword of Damocles in Kidney Transplantation for Primary Focal Segmental Glomerulosclerosis

**DOI:** 10.3389/fimmu.2019.01669

**Published:** 2019-07-17

**Authors:** Katrin Kienzl-Wagner, Siegfried Waldegger, Stefan Schneeberger

**Affiliations:** ^1^Department of Visceral, Transplant and Thoracic Surgery, Medical University of Innsbruck, Innsbruck, Austria; ^2^Department of Pediatrics, Medical University of Innsbruck, Innsbruck, Austria

**Keywords:** focal segmental glomerulosclerosis, primary FSGS, kidney transplantation, recurrence, recurrent FSGS, management, allograft transfer

## Abstract

A major obstacle in kidney transplantation for primary focal segmental glomerulosclerosis (FSGS) is the risk of disease recurrence. Recurrent FSGS affects up to 60% of first kidney grafts and exceeds 80% in patients who have lost their first graft due to recurrent FSGS. Clinical and experimental evidence support the hypothesis that a circulating permeability factor is the mediator in the pathogenesis of primary and recurrent disease. Despite all efforts, the causing agent has not yet been identified. Several treatment options for the management of recurrent FSGS have been proposed. In addition to plasma exchange, B-cell depleting antibodies are effective in recurrent FSGS. This indicates, that the secretion and/or activity of the postulated circulating permeability factor(s) may be B-cell related. This review summarizes the current knowledge on permeability factor(s) possibly related to the disease and discusses strategies for the management of recurrent FSGS. These include profound B-cell depletion prior to transplantation, as well as the salvage of an allograft affected by recurrent FSGS by transfer into a second recipient.

## Key Points

1. Correct assigning of FSGS to its subclasses primary, genetic, and secondary is pivotal for therapeutic decisions and assessment of recurrence risk after renal transplantation.
2. It is obvious that one or possibly more circulating permeability factors are causative in the pathogenesis of primary FSGS and its recurrence.
3. This circulating permeability factor(s) has not yet been identified.
4. Additionally, B cells may play a central role in the pathogenesis of FSGS and its recurrence. This hypothesis is supported by the success of plasma exchange and B-cell depleting antibodies (rituximab, ofatumumab) in the treatment and pretreatment of FSGS recurrence. However, CD20-directed antibodies might also exert a beneficial effect by stabilizing podocyte function after directly binding to podocyte-derived antigens.
5. Allograft transfer of a kidney transplant that failed in the first recipient due to fulminant FSGS recurrence into a second recipient is an option to rescue the allograft and to avoid futility of organs.

## Introduction

**Figure 1:**
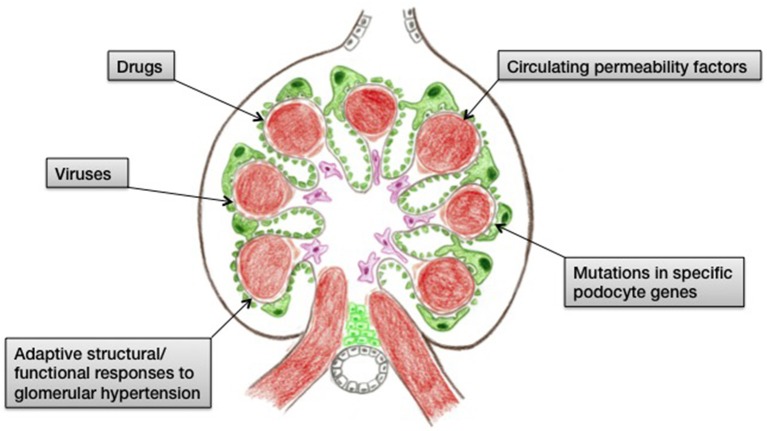
Cartoon summarizing the causes of FSGS.

Focal segmental glomerulosclerosis (FSGS) is an important cause of end-stage renal disease worldwide. It is a histologic description of a glomerular lesion caused by a multitude of pathogenicities that are linked by podocyste injury and depletion. Subclasses of FSGS include primary, genetic, and secondary forms. Genetic (or familial) FSGS is linked to specific mutations in key podocyte molecules, whereas secondary FSGS is caused by a variety of injuries such as drugs (heroin, bisphosphonates, anabolic steroids, CNIs, mTOR inhibitors, interferon), infections (HIV, parvovirus B19, CMV, EBV, SV40), or the mal-adaptive alterations that occur after any loss of kidney parenchyma (Figure 1) (1–5). In contrast to familial and secondary FSGS, primary (or idiopathic) FSGS is presumably caused by a circulating factor, possibly a cytokine elaborated from extrarenal sources, which causes generalized injury to podocytes. Primary FSGS may respond to corticosteroids, immunomodulatory agents, plasmapheresis, or immunoadsorption, and is prone to recur post transplantation.

The incidence of primary FSGS recurrence after kidney transplantation varies between 40 and 60% (6–8). Diagnosis of recurrent FSGS involves new-onset progressive proteinuria post-transplant. The only initial finding in allograft biopsies is diffuse podocyte foot process effacement on electron microscopy. Risk factors for disease recurrence include young age at disease onset (i.e., pediatric patients), rapid progression to end-stage renal disease, bilateral nephrectomy, white race, and loss of a previous allograft due to FSGS recurrence (9–11). Serum albumin at time of FSGS diagnosis (<25 g/l) is a negative predictor of FSGS recurrence (6). In kidney retransplantation for primary FSGS, recurrence occurs in up to 86% (8).

Compared to transplant for other causes, patients with FSGS have a significantly inferior graft survival with a 5-year graft survival of 81% in FSGS recipients vs. 88% in non-FSGS recipients. In pediatric patients, the difference in the transplant outcome is even more pronounced with 68% 5-year graft survival in FSGS recipients compared to 93% in non-FSGS recipients. FSGS recurrence may be fulminant with nephrotic-range proteinuria appearing hours to days after transplantation. Most commonly, recurrence occurs in the first 2 years following kidney transplantation (12). For patients with FSGS, disease recurrence is a strong predictor for graft outcome. The 5-year graft survival in patients with FSGS recurrence is 52% compared to 83% in patients without FSGS recurrence. Recurrence leads to graft loss in half of the patients within 5 years. The majority of graft losses occurs during the first 2 years after disease recurrence (6, 7, 12). The incidence of delayed graft function seems to be higher in FSGS patients with disease recurrence compared to patients without recurrence. Interestingly, patients with FSGS recurrence experience significantly more biopsy-proven acute rejections than patients without recurrence (6, 13).

An analysis of the North American Pediatric Renal Transplant Cooperative Study (NAPRTCS) data revealed that the expected graft survival advantage of a live donor kidney transplant in patients with primary FSGS was lost due to the increased risk for FSGS recurrence (13). A more recent retrospective analysis of the UNOS registry including more than 2,000 pediatric kidney transplants for primary FSGS found that the donor type in pediatric patients was not independently associated with disease recurrence (11). Using the Australian and New Zealand Dialysis and Transplant (ANZDATA) registry, Francis et al. demonstrated a weak, albeit significant association between donor type and FSGS recurrence with a higher recurrence rate in recipients of a live donor transplant. Live donor kidney transplantation for FSGS resulted in significantly improved graft survival with a median graft survival of 14.8 years in recipients of a live donor transplant compared to 12.1 years for recipients of a deceased donor transplant. Importantly, a survival advantage was also observed in the pediatric population. A 5-year allograft survival of 80% after living donor kidney transplantation compares favorably to 46% in deceased donor organ recipients (12). Based on these data, kidney transplantation for truly idiopathic FSGS should not preclude the use of a suitable living donor.

## Disease Pathogenesis

There is clinical and experimental evidence that a circulating permeability factor is involved in the pathogenesis of primary FSGS and its recurrence. The first clinical evidence for a causative circulating factor dates back to 1972 when Hoyer et al. reported 3 patients who experienced recurrence of FSGS after kidney transplantation despite profound immunosuppressive therapy (14). Additional clinical evidence is derived from a case report of transmission of the circulating permeability factor from mother to child during pregnancy (15). The hypothesis of a causative circulating factor in disease pathogenesis is further supported by the apparent effect of postoperative plasma exchange on proteinuria in recurrent FSGS (16). Plasmapheresis also has a preemptive effect toward disease recurrence in high risk renal transplant recipients (17). Further, the successful transplantation of a renal allograft that failed in the first recipient due to fulminant FSGS recurrence into a second recipient corroborates the causative role of circulating factor(s) in primary and recurrent FSGS (18, 19). Experimental findings which support the hypothesis of a circulating permeability factor include the induction of proteinuria in isolated rat glomeruli after injection of serum from patients with primary FSGS (20).

The evolution of the search for permeability factors has been reviewed by Maas et al (21). The first experimental studies on vascular permeability factors in nephrotic syndrome date back to the 1970s (22, 23). Experimental models to study permeability factors in recurrent FSGS include the *in vitro* model of isolated rat glomeruli presented by Savin et al. (24). Preincubation of isolated rat glomeruli in serum of patients with recurrent FSGS resulted in less glomerular swelling due to loss of glomerular permeability and dissipation of the oncotic gradient when compared with healthy control sera. Thus, the oncotic pressure gradient serves as a surrogate parameter for the maintenance or the loss of the glomerular permeability barrier in healthy and diseased samples, respectively, and is therefore thought to indicate the effect of a putative FSGS permeability factor. Other experimental studies have used podocyte cell cultures to study the direct effects of FSGS circulating factors on podocytes (25, 26).

Despite decades of experimental research the postulated permeability factor in FSGS has not yet been identified. The most prominent and most extensively studied candidate factor is soluble urokinase-type plasminogen activator receptor (suPAR). Patients with FSGS were shown to have increased serum concentrations of suPAR (27). The proposed effects of suPAR include alteration of podocyte cytoskeleton, altered podocyte attachment with activation of β3-integrin, and activation of STAT1 in vascular smooth muscle cells via a PDGF receptor. Elevated suPAR concentrations however are not specific for (recurrent) FSGS but rather are associated with progression of renal disease regardless of its etiology and are partly due to diminished loss in urine (21, 28). Hence the experimental findings concerning the causative role of suPAR in FSGS pathogenesis remain inconsistent.

Another candidate protein that has been proposed to play a role in FSGS is cardiotrophin-like cytokine factor 1 (CLCF-1) (20). CLCF-1 is a member of the interleukin-6 cytokine family and acts through a complex receptor composed of CNTFR (ciliary neutrotrophic factor receptor), LIFR (leukemia inhibitory factor receptor), and gp130 (glycoprotein 130). Receptor-ligand binding initiates signaling via the JAK/STAT (janus kinase/signal transducer and activator of transcription) pathway thereby altering podocyte actin cytoskeleton. The increases of glomerular permeability in isolated rat glomeruli induced by FSGS sera and CLCF-1 were comparable in magnitude and were each inhibited by a monoclonal antibody to CLCF-1 and specific JAK inhibition (28–30).

Maas et al. have proposed four criteria that are needed to evaluate the pathogenic and causative role of a putative disease-causing permeability factor: (1) The permeability factor must have biologic effects *in vitro* and *in vivo*, and be confirmed in validation studies. (2) The permeability factor must be identified in well-phenotyped patients but not in appropriate controls and validated in independent patient cohorts. (3) The temporal relation of the permeability factor with disease activity and remission has to be demonstrated. (4) Specific removal or inhibition of the permeability factor *in vivo* must block the biologic effect (21).

Applying an integrative bioinformatics approach, Delville et al. identified autoantibodies against a panel of nearly 800 unique antigens that were significantly increased in the sera of patients that developed FSGS recurrence after transplant compared to a smaller number of elevated antibodies (*n* = 78) in the non-recurrent group. The differing antibody profiles in sera of patients with recurrent vs. non-recurrent FSGS demonstrate that FSGS is associated with a signature of increased humoral response to a variety of non-HLA antigens. A biomarker panel of 7 antibodies targeting glomerular antigens can predict post-transplant FSGS recurrence with 92% accuracy. In this panel, the anti-CD40 antibody exerts the strongest correlation with FSGS recurrence prediction. Immunohistochemistry showed positive staining for CD40 in podocytes from FSGS whereas there was no CD40 protein expression in normal renal tissue. Furthermore, anti-CD40 antibodies isolated from recurrent FSGS patients induced podocyte depolarization and blocking CD40 relieved proteinuria (31).

Clinical and experimental research has proposed several candidates for the postulated circulating permeability factor in FSGS. Although the causative role could not be validated for any of these candidates yet, it seems highly likely that one or more circulating permeability factors are causative in disease pathogenesis and recurrence.

## Management of Recurrent FSGS

The management of recurrent FSGS following kidney transplantation remains extremely challenging. Due to lack of randomized trials, treatment recommendations are based on small study cohorts, case series, and case reports. Interpretation of the published literature is hindered by the heterogeneity regarding the diagnosis of primary FSGS (truly idiopathic, exclusion of genetic causes, availability of renal biopsies, HE histology, and electron microscopy), disease recurrence (clinical parameters, availability of HE histology, and electron microscopy), and definition of remission. There is no established standard for the treatment of recurrent FSGS post-transplant. Instead, various therapeutic regimens are applied (variability in initiation of treatment, number of apheresis sessions, timing and dosage of depleting antibodies, concomitant immunosuppressive regimens, etc.), as recurrence of FSGS post-transplant is associated with accelerated graft loss. Clinical decision making is often driven by the fear of loss of the allograft. The application of several treatments simultaneously is favored over establishing hypothesis-driven therapies and controlled trials.

**Figure 2:**
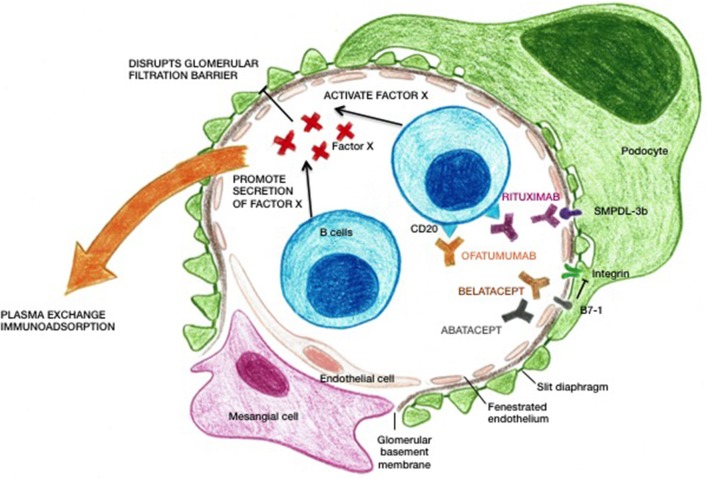
Scheme illustrating the therapeutic targets in recurrent FSGS. Factor X denominates the yet unidentified circulating permeability factor(s).

The therapeutic targets of the different treatments that are currently applied in recurrent FSGS are illustrated in Figure 2.

### Plasma Exchange

Building on the assumption that a possible serum factor was responsible for FSGS disease and recurrence, Zimmermann was the first to report plasma exchange as a treatment option for FSGS recurrence in 1985 (16). Plasma exchange replaces the patient's blood plasma with donor plasma, thereby removing potential pathological factors from the patient's circulation. Current guidelines recommend the use of plasma exchange for recurrent post-transplant FSGS, although there are no controlled trials assessing the efficacy of plasma exchange compared to alternative treatments or no treatment.

A recent systematic review by Kashgary et al. identified 43 case series and 34 case reports of recurrent FSGS treated with plasma exchange (32). Of the 423 patients with recurrent post-transplant FSGS, 47% achieved complete remission (defined as proteinuria <0.5 g/day) after treatment with plasma exchange, and 28% had partial remission (defined as proteinuria between 0.5 g and 3.5 g/day). Remission was similar for adults and children (69 and 70%, respectively). Males were nearly three times as likely to achieve remission as females (odds ratio 2.85), and patients who received treatment within 2 weeks of recurrence were twice as likely to achieve remission when compared to patients with delayed initiation of treatment (odds ratio 2.16), although this effect did not reach statistical significance. Patients with proteinuria >7 g/day at recurrence were less likely to achieve remission (odds ratio 0.43). Age and type of transplant were not associated with remission. The overall remission rate of 71% appears favorable compared to historical controls with reported remission rates of <30%.

### Immunoadsorption

In contrast to total plasma exchange, immunoadsorption (IA) is a selective apheretic procedure that does not induce loss of coagulation factors and therefore might be preferable with respect to postoperative bleeding risk. Immunoadsorption can either be performed with a protein A column or an IgG column. Immunoadsorption for the treatment of relapsing FSGS following kidney transplantation was originally described by Dantal et al. (33). In a recent multicenter cohort of 12 pediatric patients treated with immunoadsorption for FSGS recurrence post-transplant, Allard et al. reported partial or complete remission in 10 children. Decrease of proteinuria occurred rapidly within the first 10 sessions of immunoadsorption. Even though IA was safe and effective, the effect of immunoadsorption was not lasting and hence requiring chronic IA to maintain remission (34). Given the published experience, IA can be considered a therapeutic option in recurrent FSGS due to its more selective performance. Nevertheless, its clinical value is greatly impacted by its transient effect.

### Rituximab

Rituximab is a chimeric mouse/human monoclonal antibody that targets CD20 on B-cells thereby inducing selective and profound B-cell depletion for 6 months following antibody administration. Apart from this immunological effect, rituximab is thought to act as a direct modulator of podocyte function. Through binding to sphingomyelin-phosphodiesterase-acid-like-3b (SMPDL-3b) on podocytes, rituximab exerts a protective effect on the podocyte actin cytoskeleton (35, 36).

The beneficial effect of rituximab in recurrent FSGS was first described by Pescovitz (37). Recurrent FSGS in a pediatric patient resolved only after his post-transplantation lymphoproliferative disease (PTLD) was treated with rituximab, suggesting that B-cells might play a central role in FSGS disease and recurrence. Alternatively, protective effects of rituximab-binding on podocytes might explain the induction of disease remission. Since then, several retrospective case series have reported encouraging results for the use of rituximab in recurrent FSGS [summarized in Cravedi et al. (38)]. In their recent retrospective multi-center cohort study, Garrouste et al. investigated the effect of rituximab introduced either immediately after FSGS recurrence in combination with plasma exchange or after failure of plasma exchange and rituximab administered for failed attempted weaning from plasma exchange (39). Complete and partial remission was achieved in 47 and 16% of patients, respectively. At 12 months following rituximab treatment, eGFR was significantly lower in patients not responding to rituximab (25 ± 20 vs. 43 ± 20 ml/min/1.73 m^2^). Five-year allograft survival was 100% in responding compared to 34.3% in the non-responding patients.

### Ofatumumab

Ofatumumab is a human anti-CD20 monoclonal antibody that binds to an epitope of CD20 distinct from the rituximab binding site. It induces profound B-cell depletion for up to 6 to 10 months after therapy. With ofatumumab, complement dependent cytotoxicity (CDC) appears to be greater and occurs even at a lower density on the cell surface than with rituximab. The greater binding avidity to CD20 is thought to be responsible for improved CDC (40).

Basu et al. observed a dramatic improvement in renal function in a patient with rituximab-resistant FSGS following therapy of chronic lymphocytic leukemia with ofatumumab. Ofatumumab was shown to be effective in four more patients with rituximab-resistant nephrotic syndrome and led to a decline in the protein-creatinine ratio and a rise in estimated glomerular filtration rate at 6 months (41).

Wang et al. (42) and Bernard et al. (43) were the first to report the successful use of ofatumumab in the setting of post-transplant FSGS recurrence refractory to rituximab treatment. Ofatumumab was introduced 2.5 years and 8 months after transplantation, respectively, and resulted in remission of nephrotic-range proteinuria and hence stabilization of renal allograft function.

### Abatacept and Belatacept

Abatacept (cytotoxic T-lymphocyte-associated antigen 4-immunoglobulin fusion protein CTLA4-Ig) is an inhibitor of the T-cell costimulatory molecule B7-1 (CD80). B7-1 is not expressed in normal human kidney podocytes but is found in biopsy specimen from patients with recurrent FSGS. Clinical and *in vitro* data from Yu et al. (44) indicate that B7-1 mediates podocyte injury and proteinuria by blocking β1-integrin activation, therefore promoting disease-associated podocyte migration. Abatacept protects β1-integrin activation in podocytes which is thought to be the underlying mechanism for the antiproteinuric action of abatacept. Using abatacept, the authors successfully induced complete or partial proteinuria remission in four patients with recurrent FSGS.

These promising results were refuted by the study of Delville et al. who prospectively administered abatacept or belatacept (a B7-1 blocker with a 2-fold higher affinity for B7-1) to nine consecutive patients with recurrent FSGS after transplant. Neither did abatacept/belatacept induce proteinuria remission, nor did the authors detect podocyte B7-1 expression in the biopsies performed at the time of FSGS recurrence (45). Accordingly, Grellier et al. (46) and Alachkar et al. (47) did not have positive therapeutic responses of B7-1 blockade in recurrent FSGS.

### Kidney Retransplantation

In patients who have lost their first allograft due to recurrent FSGS, kidney retransplantation is associated with recurrence rates of 86% (8). In the light of organ shortage, such a high recurrence risk may be a barrier for relisting these patients for a second kidney transplant. Living donor kidney transplantation may even be considered unethical.

The concept of an allograft removal and transfer to another recipient in the setting of fulminant FSGS recurrence after kidney transplantation was first described by Gallon et al (18). The authors reported the case of a 27-year old patient with primary FSGS who developed fulminant disease recurrence on day 2 after a living donor kidney transplant. Despite plasma exchange, the allograft did not recover. Due to persistent proteinuria, hypoalbuminemia and the development of intraabdominal hematoma, the allograft did not function and had to be removed on postoperative day 14. The allograft was immediately retransplanted into a 66-year old second recipient with end-stage renal disease due to type 2 diabetes mellitus. The allograft function returned soon after retransplantation and kidney biopsies after retransplantation showed reversal of the histopathological lesions (podocyte foot-process effacement and loss of the interdigitating arrangement). Eight months after transplantation, the recipient had excellent graft function with a glomerular filtration rate above 90 ml/min and only mild proteinuria.

Building on this case, our group just recently reported the second case of successful allograft transfer after FSGS recurrence in the transplant (19). A 5-year old boy with primary FSGS received a deceased donor renal transplant from a 31-year old donor. Despite initial graft function, the boy developed fulminant recurrence of FSGS on day 2 post transplant. Rituximab and a series of plasmapheresis did not show any effect. Repeat renal biopsies excluded rejection or severe damage of the graft despite anuric graft failure. To avoid immunosuppression-associated complications upon restart of peritoneal dialysis, graft nephrectomy on day 27 after kidney transplantation was planned. After informed consent by the family was given, the allograft was retransplanted into a 52 year old recipient with vascular nephropathy on the waiting list. Immediately after retransplantation, the allograft regained function with serum creatinine levels decreasing to 0.8 mg/dl and proteinuria decreasing to 0.22 g/g at hospital discharge. Three years after retransplantation, the second recipient continues to have excellent graft function with serum creatinine levels of 0.9 mg/dl and clinically insignificant proteinuria (<0.2 g/g).

These two cases give evidence that in the setting of FSGS recurrence, retransplantation of the failed allograft into a second recipient can be applied to organs from living as well as deceased donors with a time frame between transplantation and allograft transfer of 4 weeks, possibly even longer. The strategy of allograft transfer may ease the decision to put a patient on the waiting list who has experienced FSGS recurrence in the first transplant and therefore carries an extremely high risk of disease recurrence and hence graft failure after kidney retransplantation. Given adequate counseling of both donor and recipient, the concept of allograft transfer also justifies living donation for kidney retransplantation after failure of the first allograft due to FSGS recurrence.

### Pre-conditioning Regimens for Transplantation in Primary FSGS Patients

In patients who are deemed high risk for recurrence after kidney transplantation, pretreatment of the recipient to prevent post-transplant FSGS recurrence has been proposed. Based on the hypothesis that removing the putative circulating permeability factor(s) before and after renal transplantation will ameliorate the course of FSGS, Ohta et al. were the first to describe preoperative plasmapheresis as a preconditioning regimen for transplant in FSGS patients (48). Prophylactic plasmapheresis was performed on days 5, 3, and 1 prior to living donor kidney transplantation. In case of FSGS recurrence, therapeutic plasmapheresis was continued until proteinuria was markedly reduced or disappeared. Employing this approach, the authors observed a 50% reduction of FSGS recurrence.

In a recent prospective observational cohort study, Alasfar et al. assessed the efficacy of perioperative rituximab and therapeutic plasma exchange for preventing post-transplant FSGS recurrence (49). Based on their assumed risk of recurrence, patients received a preemptive therapy with either rituximab alone or rituximab and plasma exchange or no pretreatment. Rituximab was given in one or two doses (375 mg/m^2^ per dose), and perioperative plasma exchange sessions were started between day 7 before transplant and postoperative day 2. Plasmapheresis was then continued for a total of 3 to 10 sessions. The authors concluded that there was no difference in recurrence rates between patients who received a preemptive therapy and those who did not receive a pretreatment (62 and 51%, respectively). The major limitation of this study is that only patients with at least two risk factors for disease recurrence received preemptive treatment. The observed recurrence rates therefore need to be interpreted with caution. Nevertheless, the study stands out as one of the largest prospective trials with a highly selected study cohort of truly idiopathic FSGS patients including 36% of patients receiving a kidney retransplant (second, third, or fourth renal transplant) and involving both deceased and living donor kidney transplants.

In addition to plasma exchange, it has been postulated that LDL (low-density lipoprotein) apheresis eliminates humoral factors. Accordingly, Sannomiya et al. applied pretransplant LDL apheresis in 5 patients with FSGS to prevent disease recurrence (50). The pretreatment regimen in these patients consisted of rituximab (100 mg), tacrolimus, mycophenolate mofetil, and methylprednisolone on day 4 before transplantation. LDL apheresis was initiated on days 3 and 1 before transplantation. Basiliximab (20 mg) was given intraoperatively and on day 4 after transplantation. During the short observation period (2 to 22 months) the authors did not observe recurrence of FSGS in all 5 patients receiving preoperative LDL apheresis.

Building on recent case reports about the successful use of ofatumumab in the treatment of post-transplant FSGS recurrence, our group hypothesized that pretreatment of the recipient with ofatumumab before a second kidney transplant could prevent FSGS recurrence through induction of profound B-cell depletion prior to the transplant (19). Following graft loss due to fulminant FSGS recurrence, the 8-year old patient with idiopathic FSGS was put on the waiting list for a second kidney transplant. He received ofatumumab at a dose of 175 mg/m^2^ during week 1, followed by 1,150 mg/m^2^ weekly for 5 weeks. Four months later, the boy received a deceased donor renal transplant from a 2-year old DCD (donation after circulatory death) donor (category III). Moreover, to prevent FSGS recurrence, daily plasma exchange was initiated immediately after transplant. Despite excellent allograft function with normal serum creatinine levels, severe proteinuria indicated FSGS recurrence within days after the transplant. A series of 15 plasma exchanges and administration of ofatumumab (1,150 mg/m^2^) on day 33 post-transplant reduced proteinuria to subnephrotic levels. In response to an increase in B lymphocytes with a concomitant rise in urinary protein levels at 6 months after transplantation, ofatumumab was administered at a dose of 1,150 mg/m^2^ every 6 months. This reduced proteinuria to subnephrotic levels. At 17 months after kidney retransplantation, the patient has excellent graft function with a serum creatinine of 0.4 mg/dl. We feel that ofatumumab represents a novel therapeutic option not only in the treatment of manifest recurrent FSGS but also as a preemptive therapy for FSGS recurrence after kidney (re)transplantation.

## Author Contributions

KK-W wrote the manuscript. SW and SS critically revised the manuscript. SS was responsible for the overall preparation of the manuscript.

### Conflict of Interest Statement

The authors declare that the research was conducted in the absence of any commercial or financial relationships that could be construed as a potential conflict of interest.

## Abbreviations

ANZDATA: Australian and New Zealand Dialysis and Transplant
CDC: complement dependent cytotoxicity
CLCF-1: cardiotrophin-like cytokine factor 1
CMV: cytomegalovirus
CNI: calcineurin inhibitor
CNTFR: ciliary neutrotrophic factor receptor
CTLA4-Ig: cytotoxic T-lymphocyte-associated antigen 4-immunoglobulin fusion protein
DCD: donation after circulatory death
EBV: Epstein Barr Virus
eGFR: estimated glomerular filtration rate
FSGS: focal segmental glomerulosclerosis
gp130: glycoprotein 130
HIV: human immunodeficiency virus
IA: immunoadsorption
JAK: janus kinase
LIFR: leukemia inhibitory factor receptor
LDL: low-density lipoprotein
mTOR: mammalian target of rapamycin
NAPRTCS: North American Pediatric Renal Transplant Cooperative Study
PDGF: platelet derived growth factor
PTLD: post-transplantation lymphoproliferative disease
SMPDL-3b: sphingomyelin-phosphodiesterase-acid-like-3b
STAT1: signal transducer and activator of transcription 1
suPAR: soluble urokinase-type plasminogen activator receptor
SV40: Simian Virus 40
UNOS: United Network for Organ Sharing.
